# Continuous Positive Airway Pressure Effects on Pulmonary Hemodynamics and Left Ventricular Function in Obstructive Sleep Apnea and Obesity Hypoventilation Syndrome: A Systematic Review and Meta‐Analysis

**DOI:** 10.1155/carj/3020878

**Published:** 2026-10-12

**Authors:** Jing Li, Jing Zhao, Pan-Pan Pang, Wei-Jie Zhai, Ya-Kun Wang, Xiao-Ying Zhang, Yu-Qing Wei

**Affiliations:** ^1^ Department of Respiratory and Sleep Medicine Hebei Medical University Third Hospital Shijiazhuang 050051 Hebei China

**Keywords:** continuous positive airway pressure, left ventricular function, obesity hypoventilation syndrome, obstructive sleep apnea, pulmonary hypertension

## Abstract

**Background:**

Obstructive sleep apnea (OSA) and obesity hypoventilation syndrome (OHS) are strongly linked to pulmonary hypertension and left ventricular abnormalities, driving cardiovascular issues. OHS, with daytime hypercapnia, affects 10%–20% of OSA patients. Continuous positive airway pressure (CPAP) is the first‐line therapy, but its cardiac effects are inconsistent.

**Methods:**

We conducted a systematic review and meta‐analysis adhering to the PRISMA 2020 statement. Risk of bias was assessed using RoB 2 (RCTs) and the Newcastle–Ottawa Scale (NOS; cohort studies). Continuous outcomes were pooled as mean differences (MDs) with 95% CIs using random‐effects models (restricted maximum likelihood) with Hartung–Knapp adjustment.

**Results:**

Eight studies (574 participants; 2 RCTs, 6 cohorts) were included, showing that CPAP significantly reduced pulmonary arterial pressure (MD = −2.32 mmHg, 95% CI: −3.88 to −0.76, *p* = 0.004) versus controls. Secondary analyses indicated improved left ventricular diastolic function (early‐to‐late diastolic mitral inflow velocity [E/A] ratio: MD = 0.29, 95% CI: 0.09 to 0.48, *p* = 0.004). No significant effects on left ventricular mass index (LVMi) (MD = −2.31 percentage points, 95% CI: −6.38 to 1.77, *p* = 0.27) or left ventricular ejection fraction (LVEF) (MD = 0.53 percentage points, 95% CI: −1.49 to 2.55, *p* = 0.61) were found. Sensitivity analyses confirmed robustness. Funnel plot analysis suggested potential publication bias for pulmonary artery systolic pressure (PASP)/mean pulmonary artery pressure (mPAP), E/A ratio, and LVEF, while LVMi showed low bias.

**Conclusions:**

In adults with OSA or OHS, CPAP reduces pulmonary arterial pressure and improves LV diastolic function, but it does not significantly alter LVMi or ejection fraction. These findings support CPAP as a key intervention to mitigate pulmonary hemodynamic burden and diastolic dysfunction in this high‐risk population, while its potential to reverse structural cardiac remodeling requires further study.

## 1. Introduction

Obstructive sleep apnea (OSA) and obesity hypoventilation syndrome (OHS) are rising consequences of the global obesity epidemic and impose a substantial public health burden. OSA—recurrent upper‐airway obstruction with intermittent hypoxia (IH) and sleep fragmentation—affects ∼10–17% of middle‐aged adults and > 30% of people with obesity [1, 2]. OHS, defined by obesity and daytime hypercapnia in the absence of other respiratory disorders, occurs in 10%–20% of patients with moderate‐to‐severe OSA [3, 4]. Both conditions are linked to excess cardiovascular morbidity and mortality [5, 6].

Cardiovascular injury is a central complication in OSA/OHS, with pulmonary hypertension (PH) and left ventricular (LV) remodeling/dysfunction driving adverse outcomes. PH—typically defined by elevated pulmonary artery pressures (PAPs) on echocardiography or right heart catheterization (RHC)—occurs in 17%–53% of OSA and 40%–60% of OHS cohorts [7, 8]. Mechanistic pathways include IH‐induced vasoconstriction and endothelial dysfunction with vascular remodeling (augmented in OHS by chronic hypercapnia), sympathetic overactivity that increases afterload and promotes LV hypertrophy, and systemic inflammation/metabolic dysregulation contributing to diastolic dysfunction [9–13]. Clinically, PH approximately doubles cardiovascular hospitalization and raises all‐cause mortality by 30%–50% [14]; LV hypertrophy and diastolic dysfunction predict heart failure with preserved ejection fraction (HFpEF) [15].

Continuous positive airway pressure (CPAP) is the first‐line therapy for moderate‐to‐severe OSA, improving sleepiness and correcting IH per guideline recommendations [2, 3]. Experimental and observational data suggest favorable cardiovascular effects (reduced sympathetic activation, improved endothelial function, and lower incident hypertension with long‐term use) [16]. However, effects on pulmonary pressures and LV structure/function remain inconsistent. Small randomized controlled trials (RCTs) demonstrate pulmonary artery systolic pressure (PASP) reduction with effective CPAP [17], whereas limited OHS cohorts report null findings after short follow‐up [18]. Heterogeneity in study design, outcome ascertainment (echocardiographic vs. catheter‐based pressures; methods for LV mass index [LVMi]), and inadequate assessment of effect modifiers (baseline PH, phenotype, and adherence) likely contribute to discordant results [19]. For instance, a 2009 meta‐analysis focused solely on systemic blood pressure and left ventricular ejection fraction (LVEF), neglecting pulmonary hemodynamics [20].

This systematic review and meta‐analysis aims to quantify the effects of CPAP on PAP, LVMi, diastolic function (E/A ratio), and LVEF in adults with OSA or OHS, clarifying its role in cardiovascular protection.

## 2. Methods

### 2.1. Protocol and Reporting

This meta‐analysis was conducted in adherence to the Preferred Reporting Items for Systematic Reviews and Meta‐Analyses (PRISMA) 2020 statement [21]. All methodological decisions were prespecified in the protocol to minimize reporting bias.

### 2.2. Eligibility Criteria

#### 2.2.1. Study Design

We included the following: (1) RCTs (including crossover designs) and (2) prospective/retrospective cohort studies (with comparator groups). Noncomparative studies (e.g., single‐arm case series), cross‐sectional studies, case reports, animal experiments, and reviews (systematic or narrative) were excluded.

#### 2.2.2. Population

Eligible studies enrolled adults (age ≥ 18 years) with the following characteristics.

OSA: OSA was defined by the apnea–hypopnea index (AHI) ≥ 5 events/hour, confirmed by polysomnography (PSG) or Level 2 portable sleep monitoring, per the American Academy of Sleep Medicine (AASM) 2012 criteria.

OHS: OHS was defined by body mass index (BMI) ≥ 30 kg/m^2^ + daytime arterial carbon dioxide pressure (PaCO_2_) ≥ 45 mmHg, in the absence of other causes of hypoventilation (e.g., chronic obstructive pulmonary disease or neuromuscular disorders), per the American Thoracic Society clinical practice guideline [22].

To address potential clinical heterogeneity between OSA and OHS, prespecified subgroup analyses were planned based on disease phenotype (OSA‐only vs. OHS‐inclusive cohorts).

Studies focusing on special populations (e.g., pediatric patients, pregnant women, and patients with end‐stage heart/lung disease) were excluded.

#### 2.2.3. Interventions and Comparators

The intervention group received CPAP.

The comparator group received active controls (e.g., sham CPAP, uvulopalatopharyngoplasty [UPPP], or alternative PAP modes) or no PAP (untreated controls or lifestyle modification alone).

#### 2.2.4. Outcomes

Studies were required to report baseline and postintervention (or between‐group) data for at least one of the following outcomes, measured via transthoracic echocardiography (TTE) or RHC (reference standard for pulmonary pressure).

#### 2.2.5. Primary Outcome

PAP was defined as either PASP or mean pulmonary artery pressure (mPAP). For TTE‐derived PASP, we only included studies that used the tricuspid regurgitant jet velocity (continuous‐wave Doppler) with the modified Bernoulli equation [3].

#### 2.2.6. Secondary Outcomes

LVMi: LVMi was calculated via linear (Devereux formula) or volumetric TTE methods and was reported in grams per square meter.

Transmitral E/A ratio: It was assessed via pulsed‐wave Doppler (early diastolic [E‐wave] vs. atrial systolic [A‐wave] velocity) and is a marker of LV diastolic function.

LVEF: LVEF was measured via Simpson’s biplane method or M‐mode and was reported as a percentage.

#### 2.2.7. Data Availability

Studies were excluded if (1) outcome data were unextractable (e.g., only graphical trends without numerical values); (2) they reported duplicate samples (same cohort reported in multiple publications, with the largest/most recent study retained); or (3) their follow‐up duration was < 1 month (insufficient to assess cardiac structural/functional changes) [19].

#### 2.2.8. Literature Search Strategy

##### 2.2.8.1. Databases and Timeframe

We searched seven electronic databases from inception to December 2025: PubMed, Web of Science Core Collection, Scopus, Embase, CNKI, WanFang, and VIP. In addition to electronic databases, we searched ClinicalTrials.gov for unpublished trials and reviewed reference lists of included studies and relevant reviews to identify additional eligible records.

##### 2.2.8.2. Search Terms

We used a combination of MeSH terms, Emtree terms, and free‐text keywords to maximize sensitivity, focused on three domains: (1) OSA/OHS, (2) PAP interventions, and (3) cardiac outcomes (Supporting Table 1). An example search string for PubMed was as follows:((“obstructive sleep apnea”[MeSH Terms] OR “obesity hypoventilation syndrome”[MeSH Terms] OR OSA[Title/Abstract] OR OHS[Title/Abstract]) AND (“continuous positive airway pressure”[MeSH Terms] OR “noninvasive ventilation”[MeSH Terms] OR CPAP[Title/Abstract] OR NIV[Title/Abstract]) AND (“pulmonary hypertension”[MeSH Terms] OR “left ventricular hypertrophy”[MeSH Terms] OR “PASP”[Title/Abstract] OR “LVMi”[Title/Abstract] OR “E/A ratio”[Title/Abstract])).

### 2.3. Study Selection and Data Extraction

#### 2.3.1. Two‐Stage Screening

Two independent reviewers screened records in two stages.

Title/abstract screening: We excluded studies that clearly failed eligibility criteria.

Full‐text screening: We reviewed remaining records against all eligibility criteria.

Discrepancies were resolved via consensus with a third reviewer. The data extracted included the following: study characteristics (design and follow‐up), participant baseline data (age, sex, and BMI), intervention details, and outcome measures (baseline and postintervention values for PASP, LVMi, E/A, and LVEF). The screening process is visualized in Figure 1.

**FIGURE 1:**
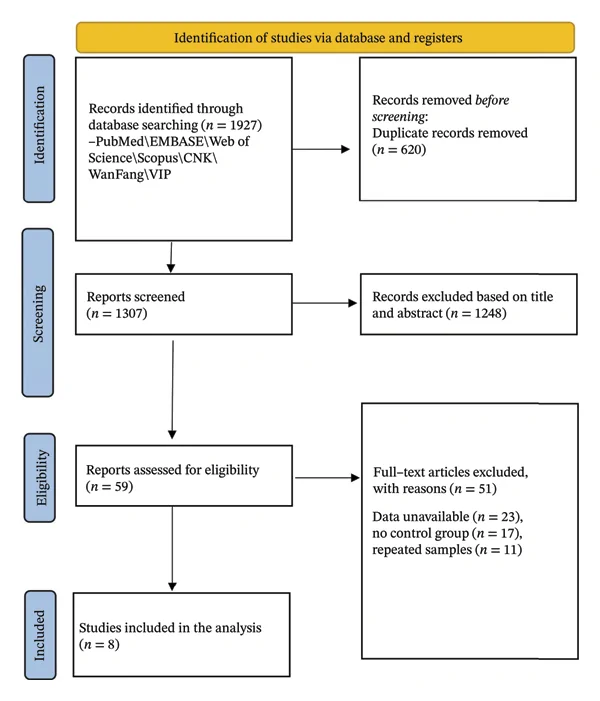
PRISMA 2020 flow diagram of literature screening and study inclusion.

#### 2.3.2. Risk of Bias Assessment

We assessed randomized trials with RoB 2 across randomization, deviations from intended interventions, missing data, measurement, and reporting domains (Arias was randomized, double‐blind, and sham‐controlled). Cohort studies were appraised with the Newcastle–Ottawa Scale (NOS) for selection, comparability, and outcome. If the risk of bias is rated as low across all domains, the overall risk of bias is judged as low.

If any domain is rated as some concerns and no domain is rated as high risk, the overall risk of bias is judged as some concerns. If any domain is rated as high risk, the overall risk of bias is judged as high risk.

### 2.4. Statistical Analysis

Given clinical/methodological diversity (designs, follow‐up durations, and PAP modes), we used random‐effects models (restricted maximum likelihood) with the Hartung–Knapp adjustment for the primary analyses. Continuous outcomes were pooled as mean differences (MDs) with 95% CIs on the original scale (mmHg for PASP/mPAP; g/*m* ^2^ for LVMi; absolute units for E/A; percentage points for LVEF). Heterogeneity was quantified with *I* ^2^ and *τ* ^2^, and we reported the 95% prediction interval when ≥ 3 studies contributed. Subgroup analyses were conducted by disease phenotype (OSA vs. OHS). To assess the robustness and stability of the pooled estimates, we performed a sensitivity analysis using the leave‐one‐out method. Sensitivity analyses excluded nonrandomized studies and used fixed‐effect models. Publication bias was assessed visually via funnel plots. All analyses were two‐sided with *α* = 0.05 and performed in RevMan5.4 (The Cochrane Collaboration, Copenhagen, Denmark) and Stata 18 (StataCorp LLC, College Station, TX, USA).

## 3. Results

### 3.1. Study Selection

As depicted in the flow diagram (Figure 1), the search identified 1927 records. After removing 620 duplicate records, 1307 titles and abstracts were screened, of which 1248 were excluded. A total of 59 full‐text articles were assessed for eligibility, and 51 were further excluded for the following reasons: only graphical abstracts without reported numerical means or standard deviations (*n* = 23), absence of a control group (*n* = 17), and repeated publication of overlapping samples (*n* = 11). Eight studies were included in the qualitative and quantitative syntheses [17, 23–29].

### 3.2. Study Characteristics

Across all included arms, 574 participants were analyzed, with follow‐up ranging from 2 months to 3 years. Baseline age across studies was approximately 51–65 years; males comprised 51%–100% of each cohort. BMI ranged from 29.0 to 43.1 kg/m^2^. Baseline pulmonary pressure indices (PASP or mPAP) ranged from 29.0 to 41.5 mmHg (Duchna, mPAP 24.1 ± 10.2 mmHg; Arias, PASP 29.8 ± 8.8 mmHg; Marvisi, PASP 39.8 ± 4.1 mmHg; Corral, PASP 38.5 ± 10.3 mmHg; Masa, mPAP 40.5–41.5 mmHg, Zhang mPAP 29.0 ± 6.6 mmHg). Full characteristics are summarized in Table 1.

**TABLE 1:** Basic characteristics of included studies.

First author (year)	Study design	Disease	Sample size (C/O)	Follow‐up duration	Age (years)	Male proportion (%)	Body mass index (kg/m^2^)	Intervention	Risk of bias
Duchna (2006) [23]	Matched cohort study	OSA	20/20	6 months	57 ± 7	100	31.2 ± 3.3	CPAP vs. untreated	NOS 8/9 points (good comparability between groups)
Arias (2006) [17]	Randomized controlled trial	OSA	23/10	12 weeks	51 ± 13	96	30.9 ± 4.0	CPAP vs. sham CPAP	Low risk of bias (randomization and allocation concealment well performed)
Zhang (2008) [24]	Prospective cohort study	OSA	7/10	6 months	51 ± 3	100	29.0 ± 1.6	CPAP vs. BiPAP	NOS 8/9 points (good comparability between groups)
Marvisi (2015) [25]	Prospective cohort study	OSA	17/8	3/6/9 months	64 ± 20	72	32 ± 6	CPAP vs. UPPP	NOS 7/9 points (complete follow‐up)
Corral (2017) [26]	Prospective cohort study	OHS	37/36	2 months	58 ± 9	52	41.8 ± 8.2	CPAP vs. NIV	NOS 7/9 points (good data integrity)
Masa (2020) [27]	Randomized controlled trial	OHS	102/94	3 years	60 (49,71)	51	42.5 (38.1, 49.1)	CPAP vs. NIV	Low risk of bias (blinded outcome assessment)
Zhang (2022) [28]	Matched cohort study	OSA	15/15	3/6/12 months	39 (31,46)	90	34.6 (29.1, 35.5)	CPAP vs. drug	NOS 8/9 points (good comparability between groups)
Ye (2023) [29]	Matched cohort study	OSA	80/80	1 year	Not reported	Not reported	29.4 (28.5, 31.1)	CPAP vs. sham CPAP	NOS 7/9 points (good data integrity)

*Note:* C: continuous positive airway pressure (CPAP), BiPAP, bilevel positive airway pressure ventilation, UPPP: Uvulopalatopharyngoplasty.

Abbreviations: CPAP: continuous positive airway pressure; NIV: noninvasive ventilation; NOS: Newcastle–Ottawa Scale; O: others; OSA: obstructive sleep apnea; OHS: obesity hypoventilation syndrome.

### 3.3. Risk of Bias

Both randomized controlled trials were judged to be at low risk of bias, with appropriate randomization, allocation concealment, and blinded outcome assessment where reported. The six cohort studies scored 7–8/9 on the NOS, indicating good methodological quality with expected limitations of nonrandomized designs. The Duchna matched‐pair cohort prespecified matching on age, sex, anthropometry, OSA severity, and antihypertensive treatment, with blinded echo assessment, supporting low selection and measurement bias on NOS items. Overall, 5/8 studies were at low risk and 3/8 at moderate risk (Figure 2; Table 1).

**FIGURE 2:**
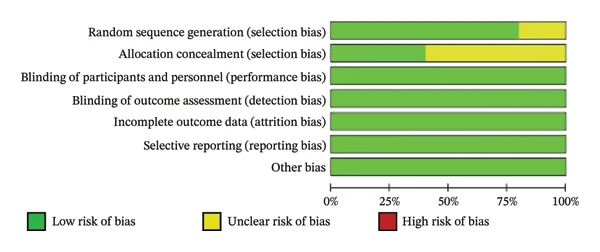
Risk of bias assessment results of inclusion studies.

### 3.4. Effects of Positive Airway Pressure on Cardiovascular Outcomes

#### 3.4.1. Pulmonary Hemodynamics (PASP/mPAP)

All 6 studies reported PAP at baseline and postintervention or versus a comparator. In random‐effects models, positive airway pressure was associated with lower pulmonary pressures compared with controls (MD, −2.32 mmHg; 95% CI, −3.88 to −0.76; *p* = 0.004) (Figure 3). Between‐study heterogeneity was evident on visual inspection of the forest plot (*I* ^2^ = 96%, *p* < 0.0001). In the subgroup analysis (Figure S1), stratified by underlying condition into OSA and OHS, positive airway pressure significantly reduced PAP in the OSA subgroup (4 studies; SMD = −2.47, 95% CI [−4.39, −0.54], *p* = 0.01), although substantial heterogeneity persisted (*I* ^2^ = 92%). Conversely, while the OHS subgroup (2 studies) showed a similar trend (SMD = −2.04), the reduction was not statistically significant (95% CI [−5.29, 1.20], *p* = 0.22) and exhibited extremely high heterogeneity (*I* ^2^ = 99%). The test for subgroup differences revealed no statistically significant variation between groups (*p* = 0.83, *I* ^2^ = 0%), suggesting that disease type may not modify the treatment effect on pulmonary pressures based on current data, though findings for the OHS group should be interpreted with caution due to the limited number of studies.

**FIGURE 3:**
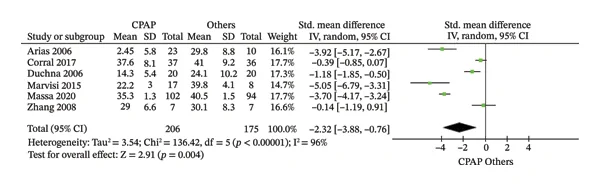
Forest plot of the effect of CPAP on pulmonary arterial pressure (PASP/mPAP).

#### 3.4.2. Diastolic Function (E/A Ratio)

Five studies contributed E/A data. Random‐effects meta‐analysis demonstrated improvement in the E/A ratio with positive airway pressure versus comparators (MD, 0.29; 95% CI, 0.09–0.48; *p* = 0.004) (Figure 4). In the subgroup analysis stratified by underlying condition (Figure S2), positive airway pressure significantly improved the E/A ratio in the OSA subgroup (3 studies; MD = 0.53, 95% CI [0.06, 0.99], *p* = 0.03), although substantial heterogeneity persisted (*I* ^2^ = 94%). Conversely, the OHS subgroup (2 studies) demonstrated a smaller but highly consistent improvement (MD = 0.03, 95% CI [0.02, 0.04], *p* < 0.00001) with no evidence of heterogeneity (*I* ^2^ = 0%). The test for subgroup differences revealed a statistically significant variation between groups (*p* = 0.04, *I* ^2^ = 77.3%), suggesting that disease type modifies the treatment effect on diastolic function, with OSA patients experiencing a greater magnitude of benefit while OHS patients exhibit a more uniform response.

**FIGURE 4:**
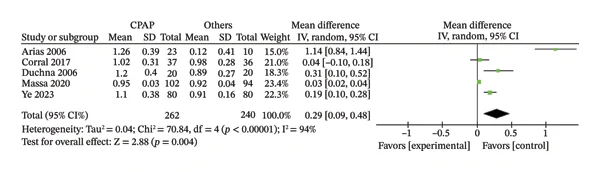
Forest plot of the effect of CPAP on transmitral E/A ratio (left ventricular diastolic function).

#### 3.4.3. LVMi

Six studies reported LVMi. Across studies reporting LVMi, there was no statistically significant difference between treatment modalities (MD, −2.31; 95% CI, −6.38 to 1.77; *p* = 0.27) (Figure 5).

**FIGURE 5:**
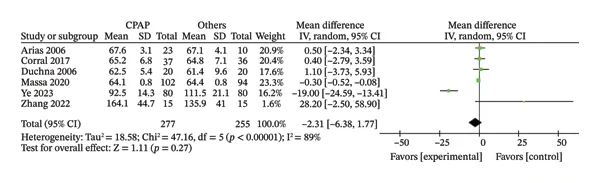
Forest plot of the effect of CPAP on left ventricular mass index (LVMi).

#### 3.4.4. LVEF

Six studies reported LVEF. Across studies reporting LVEF, there was no statistically significant difference between treatment modalities (MD, 0.53; 95% CI, −1.49 to 2.55; *p* = 0.61) (Figure 6).

**FIGURE 6:**
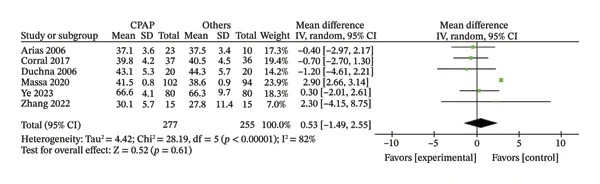
Forest plot of the effect of CPAP on left ventricular ejection fraction (LVEF).

### 3.5. Sensitivity Analysis

As illustrated in Figures S3A and 3B, the leave‐one‐out sensitivity analysis revealed that the direction of the pooled effect estimates and their 95% CIs remained unchanged after sequentially excluding each individual study. Although the point estimates fluctuated slightly and the CI widths varied modestly upon the exclusion of the studies by Ye (2023) or Corral (2017), all resulting pooled effects retained statistical significance, with no confidence intervals crossing the line of no effect. These findings indicate that the meta‐analysis results are robust and not disproportionately driven by any single study.

### 3.6. Assessment of Publication Bias

Publication bias was assessed via funnel plots for PASP/mPAP, E/A ratio, LVMi, and LVEF (Figure S4A–D), revealing varying degrees of asymmetry across outcomes. While the distribution for LVMi appeared relatively symmetric, suggesting a low risk of bias, the funnel plots for PASP/mPAP, E/A ratio, and LVEF exhibited notable asymmetry characterized by a lack of small studies with null or negative effects and the presence of outliers, indicative of potential publication bias or small‐study effects; consequently, the pooled estimates for these three parameters should be interpreted with caution.

## 4. Discussion

In this systematic review and meta‐analysis of 8 studies (*n* = 574), our meta‐analysis demonstrates that CPAP exerts favorable effects on pulmonary hemodynamics and LV diastolic function in patients with OSA/OHS, though benefits for LV structural remodeling and systolic function appear limited. However, these results must be interpreted with caution. The cardiovascular injury seen in OSA/OHS is not attributable to OSA/OHS alone; comorbid obesity, diabetes, and systemic hypertension—highly prevalent in our included cohorts—are likely to contribute substantially to the observed pulmonary vascular and LV remodeling. Whether the benefits observed here translate into hard cardiovascular outcome improvements in unselected patients remains to be established, and, if they do, the effect is likely to be concentrated in a higher‐risk subset rather than across all‐comers.

Although the absolute reduction in pulmonary pressure is numerically small, its magnitude is comparable to changes linked with clinically meaningful improvements in PH populations receiving vasodilator therapy [30]. The observed reduction in PASP is comparable to the modest hemodynamic improvements seen with oral vasodilators in mild PH populations, suggesting that CPAP offers a clinically relevant mechanical unloading effect [31]. In the context of OSA/OHS—where nocturnal upper airway obstruction provokes IH, sympathetic activation, endothelial dysfunction, and progressive pulmonary vascular remodeling—CPAP directly targets upstream drivers of increased pulmonary vascular resistance [9, 10, 16]. By stabilizing the airway and normalizing nocturnal oxygenation, CPAP likely reverses functional vasoconstriction and mitigates neurohumoral stress, thereby lowering PASP/mPAP. The concomitant improvement in the E/A ratio and reduction in LVMi are consistent with relief of afterload and sympathetic tone, remodeling of LV geometry, and better diastolic relaxation—changes that align with mechanistic data and observational signals of improved endothelial function and blood pressure control during CPAP use [16, 17]. The absence of a detectable LVEF effect is expected because the included cohorts largely had preserved baseline systolic function; in such populations, diastolic dysfunction and hypertrophy typically precede systolic impairment [15, 32].

Individual trials have yielded heterogeneous results: Short‐term randomized data in OSA showed reductions in PASP with effective CPAP [18], whereas small OHS cohorts reported null effects over brief follow‐up [19]. Prior meta‐analyses emphasized systemic blood pressure and LVEF rather than pulmonary hemodynamics or diastolic indices [33]. By focusing on PASP/mPAP, LVMi, and the E/A ratio across both OSA and OHS, our synthesis helps reconcile these discrepancies and highlights cardiopulmonary benefits beyond symptom control. Unlike prior syntheses that aggregated systemic BP or LVEF, our work uniquely isolates pulmonary vascular and diastolic responses, providing evidence that CPAP targets the specific cardiopulmonary remodeling inherent to OSA/OHS. Importantly, study‐level signals suggest greater pressure reductions among patients with baseline PH and in OHS, a phenotype characterized by more severe hypercapnia and higher pulmonary vascular load [3, 25–27]. These patterns are coherent with pathophysiology and with time‐dependent remodeling of the pulmonary circulation, where longer treatment exposure is needed to consolidate structural change. Three effect modifiers likely account for residual between‐study variability: First reason was disease phenotype (OSA vs. OHS). OHS cohorts, with higher BMI and chronic daytime hypercapnia, exhibited larger pressure reductions than OSA‐only samples in study‐level summaries [3, 26, 27]. Hypercapnia synergizes with hypoxemia to increase pulmonary vascular tone and may amplify CPAP’s hemodynamic impact when obstruction is relieved. Second reason was the treatment adherence. Studies reporting higher CPAP exposure (≥ 4 h/night) tended to show greater improvements in PASP and LVMi [2, 3, 17, 23, 27].

In our analysis, the largest improvements were consistently observed among patients with longer nightly use, with baseline PH, or with OHS—a phenotype carrying a heavier pulmonary vascular load from chronic hypercapnia. This dose–response signal supports adherence as a causal mediator of cardiovascular benefit and underscores the need for proactive adherence optimization. Accordingly, we propose that the adherence target should be risk‐adapted: For patients with known PH, LV impairment, or high hypoxic burden, a goal of ≥ 4 h/night should be emphasized; for those in good cardiovascular health with low hypoxic burden, a more flexible, symptom‐titrated approach may be appropriate to optimize the balance between therapeutic benefit and treatment burden. Finally, outcome ascertainment and analytic choices may affect the results. All included studies used TTE for PASP/LVMi. Variation in tricuspid regurgitation (TR) criteria and LV mass calculation (e.g., Devereux formula vs. volumetric methods) may introduce measurement noise [20]. While we minimized bias by applying consistent outcome definitions and excluding unvalidated protocols, TTE‐derived estimates can deviate from RHC, particularly with poor Doppler envelopes or severe TR [34]. Additionally, the small sample size limited formal meta‐regression and subgroup precision.

This study had several limitations. First, only five studies (two RCTs and three cohorts) met the inclusion criteria, constraining statistical power for subgroup analyses and limiting certainty around effect‐size heterogeneity. Second, pulmonary pressures were uniformly TTE‐derived rather than RHC‐confirmed; while TTE is practical and guideline‐concordant for screening, its accuracy varies with TR quality and loading conditions [20, 34]. Third, residual confounding cannot be excluded in observational studies (e.g., concomitant weight change, antihypertensive adjustments, and diuretic use), and adherence was not randomized. Fourth, most participants were middle‐aged men; representation of women, older adults, and patients with multimorbidity was limited, tempering external validity. Fifth, long‐term clinical endpoints (e.g., HFpEF hospitalization, atrial fibrillation incidence, and cardiovascular mortality) were not reported; thus, structural and hemodynamic benefits should be interpreted as intermediate endpoints. Finally, with only five studies, formal assessment of small‐study effects is underpowered; visual inspection did not suggest marked funnel asymmetry, but publication bias cannot be excluded. While acknowledging these limitations, we propose that the cardiovascular benefit of CPAP is likely to be most pronounced in patients with a higher pretest probability of cardiopulmonary disease—particularly those with an increased hypoxic burden or clinically significant sleepiness. These phenotypes identify subgroups in which targeted echocardiographic phenotyping and aggressive CPAP titration are most likely to be cost‐effective and clinically impactful.

Given the high prevalence of OSA/OHS and the limited availability of echocardiography in many healthcare settings, routine universal screening for PH or diastolic dysfunction is neither evidence‐based nor feasible. We propose a two‐step risk‐stratified approach to identify candidates for focused cardiovascular assessment. Step 1: Use clinically accessible indicators—moderate‐to‐severe hypoxic burden, excessive daytime sleepiness, resistant hypertension, BMI ≥ 35 kg/m^2^, or known cardiovascular disease—to flag high‐risk patients. Step 2: Perform echocardiography only in these enriched phenogroups. This strategy avoids overscreening low‐risk individuals and concentrates finite resources on those most likely to derive benefit from CPAP therapy and surveillance. Future studies should validate such a tiered model against hard endpoints.

## 5. Conclusions

Across adults with OSA or OHS, CPAP produces a modest but consistent reduction in PAP and favorable changes in LV diastolic function and mass, without altering LVEF in populations with preserved systolic function. Benefits appear greater with baseline PH, in OHS, and with sustained adherence. These findings support CPAP as a cornerstone of cardiovascular risk mitigation in sleep‐disordered breathing and outline the need for RHC‐anchored, phenotype‐aware, adherence‐targeted trials with hard clinical endpoints. Clinically, these findings support a risk‐stratified approach to echocardiographic assessment in patients with OSA/OHS and reinforce that optimizing CPAP adherence (> 4 h/night) is not merely for symptom control but is a critical strategy for preventing progression to right heart strain and diastolic heart failure.

## Author Contributions

Conceptualization, data curation, formal analysis, investigation, methodology, writing–original draft, and writing–review and editing: Jing Li, Jing Zhao, Pan‐Pan Pang, Wei‐Jie Zhai, Ya‐Kun Wang, Xiao‐Ying Zhang, and Yu‐Qing Wei.

## Funding

The authors have nothing to report.

## Ethics Statement

Since this study is based on publicly available data, no ethical approval is required for this research.

## Consent

The authors have nothing to report.

## Conflicts of Interest

The authors declare no conflicts of interest.

## Supporting Information

Additional supporting information can be found online in the Supporting Information section.

## Supporting information


**Supporting Information 1** Figure S1. Forest plot of subgroup effect of CPAP on PASP/mPAP.

**Supporting Information 2** Figure S2. Subgroup analysis stratified by underlying condition.

**Supporting Information 3** Figure S3. Sensitivity analysis of the meta‐analysis. (A) Sensitivity analysis for LVMi; (B) Sensitivity analysis for transmitral E/A ratio.

**Supporting Information 4** Figure S4. Funnel plots for the assessment of publication bias. (A) PASP/mPAP, (B) E/A ratio, (C) LVMi, and (D) LVEF.

**Supporting Information 5** Supplementary Table 1. Literature search strategy.

## Data Availability

Data sharing is not applicable to this article as no new data were created or analyzed in this study.
